# Persistent Zika virus infection in porcine conceptuses is associated with elevated *in utero* cortisol levels

**DOI:** 10.1080/21505594.2018.1504558

**Published:** 2018-08-26

**Authors:** Ivan Trus, Joseph Darbellay, Yanyun Huang, Matthew Gilmour, David Safronetz, Volker Gerdts, Uladzimir Karniychuk

**Affiliations:** a Vaccine and Infectious Disease Organization-International Vaccine Centre (VIDO-InterVac), University of Saskatchewan, Saskatoon, Canada; b Prairie Diagnostic Services, Saskatoon, Canada; c Canada National Microbiology Laboratory, Public Health Agency of Canada, Winnipeg, Canada; d Department of Veterinary Microbiology, Western College of Veterinary Medicine, University of Saskatchewan, Saskatoon, Canada; e School of Public Health, University of Saskatchewan, Saskatoon, Canada

**Keywords:** Zika virus, cortisol, pig, fetus, placenta, 11β-HSD, HPA axis

*In utero* glucocorticoid (cortisol in pigs and humans) levels are important for fetal brain development and offspring health [1,2]. It is well known that several pathogens (hepatitis B virus, cytomegalovirus, and HIV) may affect cortisol balance [3–5]. Zika virus (ZIKV)-induced pathology in symptomatic human neonates comprises small-for-gestational-age phenotype, lesions in the brain, microcephaly, epilepsy, and altered behavior associated with irritability, impatient crying, and anxiety [6]. Such clinical signs and behavioral abnormalities can be partially associated with altered glucocorticoid levels [7]. Zika virus also replicates in the placenta [8–10], a crucial player in cortisol traffic from mother to fetus in humans [11] and pigs [12]. Thus, we hypothesize that congenital ZIKV infection may affect *in utero* cortisol levels.

We have recently developed a fetal pig model which partially reproduces persistent *in utero* ZIKV infection and neonatal clinical pathology in humans [13]. Pigs share a close similarity in anatomy, growth and brain development with humans [14], and are often used in neuroscience and cortisol studies [12,14], including hypothalamic–pituitary–adrenal (HPA) axis [12,15–17] and 11β-hydroxysteroid dehydrogenase (11β-HSD) research [18–20]. Additionally, pigs can bear more than 21 fetuses with each fetus possessing an individual placenta and amniotic membranes, providing an ample number of replicates. Here, samples from our previous ZIKV studies [13] and additional challenge experiments were tested to assess *in utero* cortisol levels in amniotic fluids (AF) from conceptuses inoculated at early and midgestation.

In a recently published study [13], three selected conceptuses (fetuses with fetal membranes) from each of four experimental gilts (G296, G313 – intramniotic+ intraperitoneal (IA+IP) inoculation; G295, G314 – intracerebral (IC); a gilt is a pig pregnant for the first time) were directly inoculated *in utero* (IA+IP: 10^5^ tissue culture infectious dose with 50% endpoint (TCID_50_)/fetus, the total dose 10^5.5^ TCID_50_; IC: 10^4^ TCID_50_/fetus, the total dose 10^4.5^ TCID_50_) with a low-passage, epidemic ZIKV strain [GenBank: KU501215.1], isolated from a human sample in Puerto Rico (2015). Conceptuses from two control gilts (G270 and G312) were inoculated with virus-free media in the same manner. Inoculations were done at midgestation (50 gestation days (gd), the total duration of pregnancy in pigs is 114 days) and all inoculated and non-manipulated conceptuses and their mothers were sampled at 78 gd (36 days before birth). A detailed description of inoculation methods, sampling, and results from these animals has been published [13].

In the present study, four conceptuses from each of the two gilts were inoculated at 50 gd. Gilt G323 – two fetuses IA+IP (10^5^ TCID_50_/fetus) and two fetuses IC (10^4^ TCID_50_/fetus) with ZIKV (the total dose 10^5.3^ TCID_50_). Gilt G322 – four fetuses were inoculated in the same manner with virus-free cell culture media. However, these conceptuses and their mothers were sampled at 110 gd (four days before birth).

In addition, four conceptuses from two gilts (G27 – ZIKV, G30 – cell culture media) at early gestation (25 gd) were inoculated. At 25 gd, fetuses are too small for manipulation, and precise IP+IA/IC inoculations are not feasible. Therefore, inoculations were made into the allantoic cavity, using the ultrasound-guided technique. We used 10^6.0^ TCID_50_/fetus (10^6.6^ TCID_50_/litter) of ZIKV because abundant fluids in the allantoic cavity do not directly contact fetuses separated by amniotic membranes. Conceptuses and gilts were sampled at 110 gd following the previously published protocol [13]. Samples were tested with ZIKV specific quantitative RT-PCR, virus isolation and titration on VERO E6 cells, and histological and serological assays as previously described [13,21]. All animal experiments were approved by the University of Saskatchewan’s Animal Research Ethics Board and conducted with strickt adherence to the Canadian Council on Animal Care guidelines for humane animal use.

Amniotic fluid was collected from each conceptus using a sterile syringe and transferred to the sterile glass tube. Samples were kept on ice, aliquoted to sterile microtubes, and stored at −80°C. Maternal blood (collected from the jugular vein) was sampled in sterile EDTA tubes and centrifuged. The plasma fraction was transferred to sterile microtubes for storage at −80°C. Next, cortisol levels were measured in AF and maternal plasma (collected at the day of fetal sampling) with Cortisol ELISA Kit (ADI-900–071, Enzo Life Sciences, Farmingdale, NY, USA) according to the manufacturer’s instructions. The kit has been validated by the manufacturer for compatibility with porcine samples and has been previously used in pig research [22–25]. The assay detection limit was 56.7 pg/ml. The intra-assay coefficients of variability (CVs) were 10.5% (low), 6.6% (medium) and 7.3% (high). The inter-assay CVs were 13.4% (low), 7.8% (medium) and 8.6% (high).

Statistical analysis was performed using software GraphPad Prism 7 (GraphPad Software Inc., San Diego, CA, USA). Cortisol level and cranium dimensions between control and ZIKV groups were compared using the Mann-Whitney test. A p-value < 0.05 was considered statistically significant.

In a previous study conducted in the lab, 62 and 24 conceptuses were sampled at 78 gd from four experimental ZIKV (G295, G296, G313 and G314; exposed to ZIKV at 50 gd) and two control (G270 and G312) gilts [13]. Inoculation with ZIKV resulted in rapid trans-fetal virus spread, virus replication in fetal brains and placenta, fetal antibody (Ab) and interferon-alpha responses, and persistent *in utero* infection for 28 days. There were no apparent lesions in infected fetal brains, however, fetuses had molecular pathology in the cerebrum associated with significant dysregulation of 669 genes [13], including genes related to microcephaly, epilepsy, and embryonic brain development [13]. Samples from control gilts and all control fetuses were negative for ZIKV and specific Ab. More details regarding *in utero* infection kinetics, fetal immunological responses, and molecular pathology in fetal brains can be found in previous publication [13].

In the present study, 14 conceptuses were sampled at 110 gd (exposed to ZIKV at 50 gd) from ZIKV gilt G323 and 17 conceptuses from control gilt G322. Similar to the previous study [13], there were no significant differences in cranium dimensions between ZIKV and control fetuses (cranium diameter p = 0.87, cranium length p = 0.73; Table 1). The ZIKV litter contained two fetuses stained with meconium (**Figure S1 C;** Table 1). Meconium staining in pig and human fetuses is a sign of fetal stress which has been associated with increased cortisol levels in umbilical cord blood [26–28]. Hematoxylin/eosin staining and histopathological examination did not reveal brain lesions at 110 gd. As in previous study on pigs [13] and human studies, ZIKV RNA was detected in the placenta, amniotic membranes (AM), and AF from all directly inoculated and non-manipulated siblings (14 siblings) (Table 1). Amniotic fluid from a non-manipulated trans-infected fetuses contained infectious ZIKV reflecting productive, persistent infection for around two months (Table 1). Equivalently to human fetal infections, all ZIKV-positive porcine fetuses developed ZIKV-specific Ab suggesting previous fetal/fetal brain infection (Table 1) [13]. In contrast to the previous study where fetuses were sampled at 28 days after inoculation, ZIKV was below the detection limit in fetal brains at 60 days after inoculation (Table 1). It is possible that ZIKV in fetal brains was cleared by Ab (Table 1) and local antiviral responses [13]. Gilt G323 did not show ZIKV-specific Ab in blood plasma. Samples from control gilt G322 and all control fetuses were negative for ZIKV and Ab.

**Table 1.** Fetuses exposed to ZIKV/control media at 50 days of development and sampled at 110 days of development.

						ZIKV RNA U/g or ml					ZIKV log_10_ TCID_50_/ml	
Litter	Fetus	Fetal appearance	Cranium diameter, mm	Cranium length, mm	Cortisol in AF, ng/ml	Placenta	AM	AF	Plasma	Brain	AF	ZIKV IgG^c ^Ab log_2_ in plasma
G322 Control^a^												
	1M		53	120	na	-	-	-	-	-	-	-
	2M		49	110	21.29	-	-	-	-	-	-	-
	3F		50	110	43.59	-	-	-	-	-	-	-
	4M		48	na	16.43	-	-	-	-	-	-	-
	5F		53	110	17.40	-	-	-	-	-	-	-
	6F		54	115	44.98	-	-	-	-	-	-	-
	7F		52	105	na	-	-	-	-	-	-	-
	8F		57	115	29.66	-	-	-	-	-	-	-
	9F		52	115	52.05	-	-	-	-	-	-	-
	10F		51	115	33.41	-	-	-	-	-	-	-
	11F (IC)		54	115	40.24	-	-	-	-	-	-	-
	12 (IC)	mummification	na	na	na	-	-	na	na	-	na	na
	13F (IP+IA)		64	117	29.79	-	-	-	-	-	-	-
	14M (IP+IA)		52	115	200.00	-	-	-	-	-	-	-
	15M		54	120	27.26	-	-	-	-	-	-	-
	16M		51	114	9.12	-	-	-	-	-	-	-
	17F		47	115	101.16	-	-	-	-	-	-	-
G323 ZIKV+^b^												
	1F		53	115	198.95	-	na	2.9	-	-	-	7
	2M		53	118	82.89	2.0	5.2	4.5	0.1	-	-	9
	3M		52	110	47.56	1.8	5.7	3.8	-	-	-	9
	4M		55	120	90.71	1.9	5.6	4.8	-	-	-	7
	5M		55	112	71.97	1.9	5.1	2.9	-	-	-	7
	6F	meconium staining	52	118	62.34	4.0	5.3	3.0	-	-	-	7
	7F (IC)		51	110	61.31	-	3.3	3.3	-	-	1.5	5
	8M (IC)		50	115	na	1.7	4.6	3.3	-	-	-	5
	9F (IP+IA)	meconium staining	48	100	na	3.9	3.8	na	-	-	-	3
	10F(IP+IA)		53	110	79.36	2.4	2.5	2.8	-	-	-	3
	11F		52	115	70.74	1.1	3.9	3.4	-	-	-	5
	12F		46	105	na	3.5	2.5	3.0	-	-	-	3
	13M		50	114	na	1.3	5.9	4.3	-	-	-	7
	14M		56	125	17.48	1.3	5.3	3.9	-	-	-	5

^a^G322 Control – a control gilt; fetuses #11–14 were inoculated with virus-free media intracerebrally (IC) or intraperitoneally+intra-amniotic (IP+IA).

^b^G323 ZIKV+ – fetuses #7–10 were inoculated with ZIKV IC or IP+IA. Fetuses #6 and #9 from G323 ZIKV+ were stained with meconium (see Figure S1). Relative log_10_ TCID_50_ values were defined as RNA units (U) and expressed as ZIKV RNA U per gram (g) of tissues or milliliter (ml) of fluids [8].

^c^IgM antibodies were below the detection limit. M/F – Male/Female. AM – amniotic membrane. AF – amniotic fluid. Brain – PCR data for the cerebrum and cerebellum. “-” – below the detection limit. na – not available.

**Figure 1.**
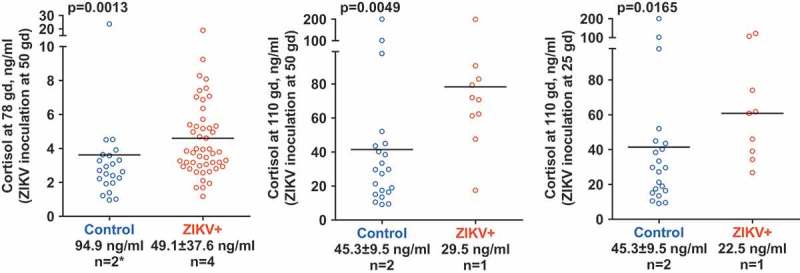
Cortisol levels in amniotic fluid of conceptuses. In accordance with physiological observations in humans [31], AF cortisol levels increased from mid (78 gd) to later gestation (110 gd) in both control and ZIKV-affected litters. Numbers below X-axis represent average cortisol level in maternal plasma and the number of tested gilts (n). Fetal cortisol data from both control gilts on middle and right panels were combined because both gilts were sampled at 110 gd. Cortisol levels in fetuses were compared using two-sided Mann-Whitney *U* test (p-value). The line represents mean value. * – at 78 gd, maternal cortisol was measured only in one control gilt.

Interestingly, all four siblings directly inoculated with ZIKV at early gestation (25 gd; gilt G27) were dead/reabsorbed at 110 gd (**Figures S1 B and D;** Table 2). In contrast, all directly inoculated and non-manipulated siblings from control gilt G30 were alive and did not have pathology or ZIKV/Ab (Table 2). As in humans [29], infection at the early stage of development may result in more severe ZIKV-induced pathology in porcine conceptuses (Table 2) compared to conceptuses inoculated at midgestation (Table 1) [13], suggesting the potential relevance of the porcine model for ZIKV pathogenesis during early pregnancy. Different routes of *in utero* inoculation at early (intra-allantoic) and midgestation (IP+IA/IC) cannot explain the observed increase in fetal mortality. In our ongoing experiments with similar or even higher ZIKV doses, intra-allantoic inoculation at midgestation does not lead to fetal death and results in similar *in utero* infection kinetics as IP+IA or IC inoculation (data are not shown). Three non-manipulated fetuses were stained with meconium (Table 2), potentially reflecting fetal stress associated with cortisol [26–28]. Cranium diameter was significantly smaller in the ZIKV-affected litter (p = 0.01, cranium length p = 0.1; Table 2). Zika virus RNA was detected in the placenta, AM, and AF from directly inoculated conceptuses and one non-manipulated adjacent sibling (Table 2). Plausibly due to the early/rapid death of directly inoculated fetuses, the virus spreads less efficiently to more distant siblings at early gestation than at midgestation (Table 1). We were not able to test ZIKV and pathology in decomposed brains from dead fetuses. Two directly inoculated fetuses showed ZIKV-specific Ab suggesting previous fetal/fetal brain infection (Table 2) [13]. Gilt G27 showed high (6 log_2_) ZIKV-specific IgG Ab titers in blood plasma indicating maternal infection.

**Table 2.** Fetuses exposed to ZIKV/control media at 25 days of development and sampled at 110 days of development.

						ZIKV RNA U/g or ml					ZIKV log_10_ TCID_50_/ml	
Litter	Fetus	Fetal appearance	Cranium diameter, mm	Cranium length, mm	Cortisol in AF, ng/ml	Placenta	AM	AF	Plasma	Brain	AF	ZIKV IgG^c^ Ab log_2_ in plasma
G30 Controla												
	1M (IA)	-	56	130	10.52	-	-	-	-	-	-	-
	2M (IA)	-	55	125	18.99	-	-	-	-	-	-	-
	3F (IA)	-	57	125	9.54	-	-	-	-	-	-	-
	4M (IA)	-	56	115	15.09	-	-	-	-	-	-	-
	5F	-	52	140	38.43	-	-	-	-	-	-	-
	6M	-	50	130	13.45	-	-	-	-	-	-	-
	7F	-	52	130	97.86	-	-	-	-	-	-	-
G27 ZIKV+^b^												
	1na (IA)	resorption	na	na	na	na	na	na	na	na	na	na
	2M (IA)	dead fetus	51	135	na	1.6	3.9	2.3	-	-	-	6
	3na (IA)	resorption	na	na	121.12	3.2	1.4	2.3	na	na	na	3^d^
	4na (IA)	resorption	na	na	na	2.3	1.5	na	na	na	na	na
	5M	meconium staining	50	120	74.05	-	1.2	< 0.1	-	-	-	-
	6F	-	52	125	34.26	-	-	-	-	-	-	-
	7F	-	49	125	46.03	-	-	-	-	-	-	-
	8M	meconium staining	43	115	39.07	-	-	-	-	-	-	-
	9F	-	50	125	60.83	-	-	-	-	-	-	-
	10M	-	54	122	26.79	-	-	-	-	-	-	na
	11M	-	50	120	106.23	-	-	-	-	-	-	-
	12F	meconium staining	51	122	61.83	-	-	-	-	-	-	-

^a^G30 Control – a control gilt; fetuses #1–4 were inoculated with virus-free media into the allantoic cavity (IA).

^b^G27 ZIKV+ – fetuses #1–4 were inoculated with ZIKV IA. Relative log_10_ TCID_50_ values were defined as RNA units (U) and expressed as ZIKV RNA U per gram (g) of tissues or milliliter (ml) of fluids [8].

^c^IgM antibodies (Ab) were below the detection limit. M/F – Male/Female. AM – amniotic membrane. AF – amniotic fluid. Brain – PCR data for the cerebrum and cerebellum. “-” – below the detection limit. na – not available.

^d^Ab were tested in AF.

Cortisol levels in AF are used to assess prenatal cortisol exposure in humans and associated developmental sequelae in offspring [30]. In this preliminary study, cortisol levels in AF from conceptuses inoculated with ZIKV at early and mid stages of development were profiled. In accordance with physiological observations in humans [31], AF cortisol levels increased from mid (78 gd) to later gestation (110 gd) in both control and ZIKV-affected litters (Figure 1). Conceptuses inoculated with ZIKV at mid and early gestation had significantly elevated cortisol levels in AF in comparison to control conceptuses (p = 0.0013–0.0164, two-sided Mann-Whitney *U* test) (Figure 1). In the present model, *in utero* ZIKV infection and maternal infection (as indicated by high titers of ZIKV-specific IgG Ab in gilt G27) did not increase cortisol levels in maternal blood, which is evident, as all ZIKV gilts had equal or lower cortisol levels than control gilts (Table 3). However, additional experiments are required to clarify the relationship between ZIKV infection and maternal cortisol levels.

**Table 3.** Cortisol level in maternal blood plasma.

Inoculation and sampling	Cortisol level in maternal blood plasma	
	Control animals, ng/ml (animal identification)	ZIKV-exposed animals, ng/ml (animal identification)
Inoculation at 50 gd, sampling at 78 gd	94.9 (G312)	11.8 (G314)
	na (G270)	29.8 (G296)
		56.5 (G295)
		98.2 (G313)
Inoculation at 50 gd, sampling 110 gd	38.6 (G322)	29.5 (G323)
Inoculation at 25 gd, sampling 110 gd	52.0 (G30)	22.5 (G27)

ZIKV – Zika virus

gd – gestation days

na – not available.

Tropism to the brain and placental cells is most likely the primary factor leading to ZIKV-induced lesions in the fetal brain and abnormal birth weight in human and porcine newborns [13,32]. However, excessive glucocorticoid exposure during *in utero* development may also lead to reduced fetal head circumference and low birthweight [2]. Moreover, animal and human studies consistently prove that excessive cortisol is neurotoxic for fetal brain development, and causes long-lasting consequences in offspring, including increased stress reactivity, cognitive/memory impairment, increased fussiness/negative behavior, mood disorder, and attention deficit-hyperactivity disorder [7]. In support of potential cortisol involvement, the altered behavior associated with irritability, impatient crying, and anxiety has been reported in human neonates affected with ZIKV *in utero* [6,32]. Neuronal depletion in fetal pig brains [33] and smaller than normal brains in porcine neonates affected with ZIKV *in utero* [13] have also been demonstrated. Importantly, in the previous study, we showed significant dysregulation of genes linked to stress responses, mood disorder, schizophrenia, and autism in brains from ZIKV-infected porcine fetuses and potentially increased activity in neonates affected with persistent ZIKV infection *in utero* [13]. Among others, NR3C1 gene, which encodes the glucocorticoid receptor related to stress and HPA axis regulation, was affected in ZIKV-infected porcine brains (false discovery rate (FDR) p-value = 0.0025; FDR < 0.05 is statistically significant) [13]. Altogether, giving clinical observations in humans, pathology in pigs, and the present cortisol data, it is possible that elevated *in utero* cortisol level during ZIKV infection may partially contribute to severe and silent fetal brain pathology, growth abnormalities in fetuses/neonates, and long-term behavioral/cognitive sequelae in offspring affected during congenital life.

To summarize, this study demonstrates that persistent ZIKV infection in porcine conceptuses is associated with elevated cortisol levels in AF. Further animal experiments and human studies on ZIKV-affected cohorts are warranted to confirm these preliminary results and clarify the potential effects of ZIKV infection on cortisol levels at different stages of gestation in mother and fetuses. It is also essential to identify how ZIKV infection impacts systems regulating *in utero* cortisol (placental 11β-HSD [11] and fetal HPA axis [1] activities), cortisol effector mechanisms (e.g. glucocorticoid/mineralocorticoid receptors in targeted tissues), and associated health sequelae in fetuses and offspring.
