# Supplementary material for: Persistent Zika virus infection in porcine conceptuses is associated with elevated in utero cortisol levels
Source: Virulence. 2018 Aug 26;9(1):1338–43. doi: 10.1080/21505594.2018.1504558 (PMC7000198; doi:10.1080/21505594.2018.1504558)
Supplement: Supplemental Material [file kvir-09-01-1504558-g0001.zip › Figure S1 caption.docx]

**Figure S1.** Healthy (**A**) and dead (**B**) fetuses from control gilt G30 and experimental gilt G27, respectively. Litters in both gilts were inoculated with control media or ZIKV at 25 gd and sampled at 110 gd. The healthy fetus is 32.7 cm (crown-rump length). The dead fetus # 2 is 23.6 cm. Meconium staining (**C**) in a non-manipulated fetus from the ZIKV-exposed litter (gilt G323, *in utero* inoculation at 50 gd, sampling at 110 gd). Resorption (**D**) of the directly inoculated fetus from the ZIKV-exposed litter (gilt G27, *in utero* inoculation at 25 gd, sampling at 110 gd).
