# Supplementary figures and images for: Persistent Zika virus infection in porcine conceptuses is associated with elevated in utero cortisol levels
Source: Virulence. 2018 Aug 26;9(1):1338–43. doi: 10.1080/21505594.2018.1504558 (PMC7000198; doi:10.1080/21505594.2018.1504558)

**A**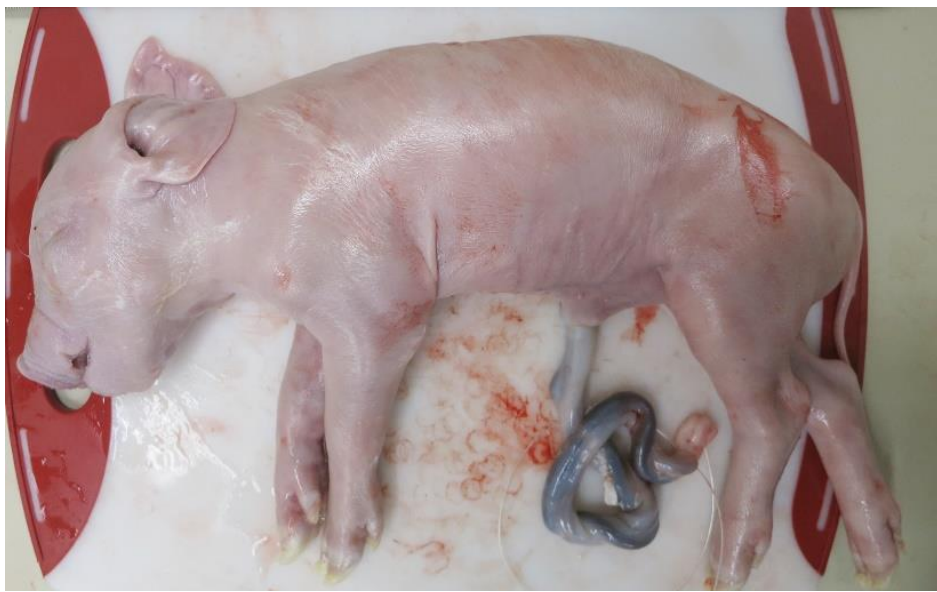**B**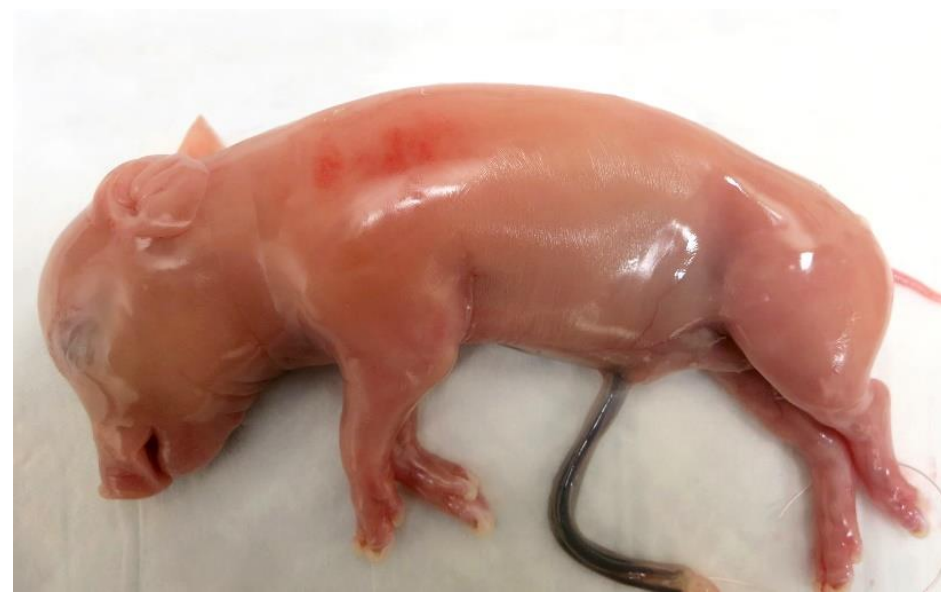**C**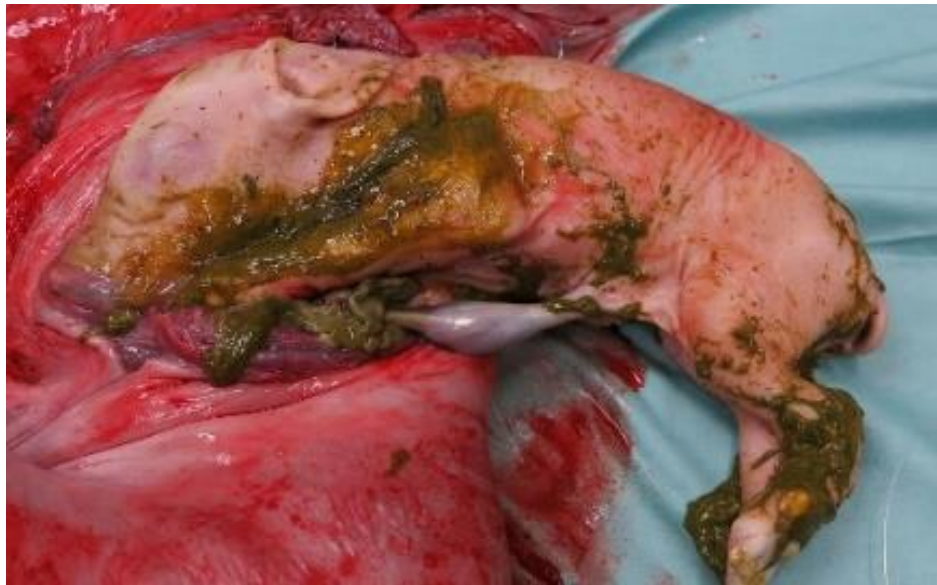**D**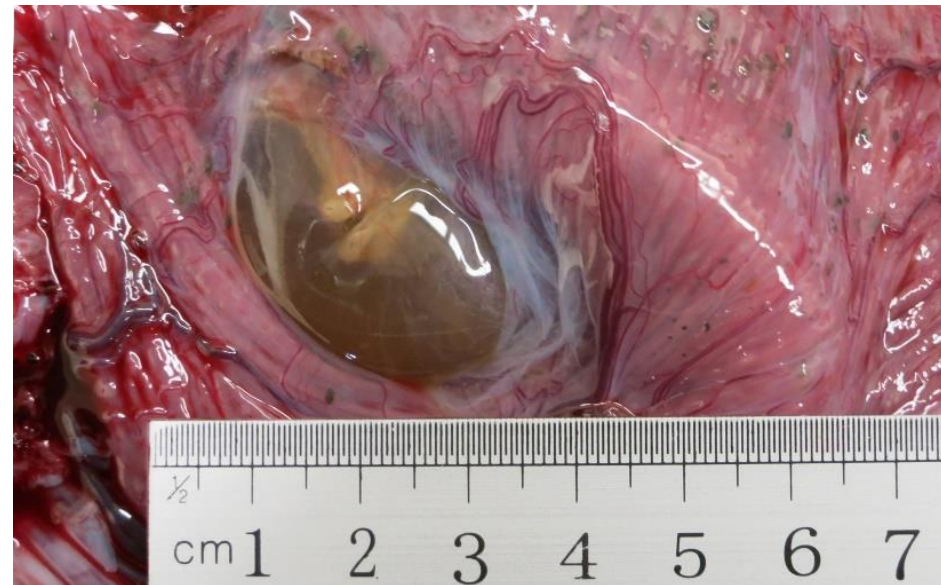

Supplement: Supplemental Material [file kvir-09-01-1504558-g0001.zip › Figure S1.pdf]
